# Website Review: Protein-Protein Interactions on the Web

**DOI:** 10.1002/cfg.111

**Published:** 2001-10

**Authors:** Jo Wixon

**Affiliations:** Bioinformatics Division HGMP-RC Hinxton, Cambridge CB10 1SB UK

## Abstract

We present a brief guide to resources on the Internet relating to Protein-Protein Interactions. These include databases containing experimentally verified and computationally inferred physical and functional interactions. There are also tools for predicting interactions and for extracting information on interactions from the literature, and organism specific databases.


**Comparative and Functional Genomics**
*Comp Funct Genom* 2001; **2**: 338–343.
**DOI:** 10.1002/cfg.111

**Feature**

### Website Review: Protein-protein interactions on the web

Jo Wixon, Managing Editor
*Bioinformatics Division, HGMP-RC, Hinxton, Cambridge CB10 1SB, UK*

### Abstract

**We present a brief guide to resources on the Internet relating to Protein-Protein Interactions. These include databases containing experimentally verified and computationally inferred physical and functional interactions. There are also tools for predicting interactions and for extracting information on interactions from the literature, and organism specific databases. Copyright © 2001 John Wiley & Sons, Ltd.**

### Introduction

How does one identify interacting proteins? Immunoprecipitation has long been used as a method for identifying those proteins that interact with a protein of choice. This approach requires antibodies directed against the protein of interest. The antibodies are used to precipitate and pull-down the protein and any other proteins that might be forming a complex with it. The unknown protein components of these complexes are commonly identified using gel electrophoresis and N-terminal sequencing or mass spectrometry. Another 'pull-down' approach involves expressing a tagged protein of interest in a cell, or mixing the purified tagged protein with cell lysate, and then using an affinity column, or beads, to purify the complexes it forms.

The yeast 2-hybrid system (Fields and Song, 1989) was initially performed using a GAL4 DNA binding domain-fusion with the chosen bait protein to capture its prey (an interacting protein) from a library of proteins labelled with the activation domain of GAL4. When the two proteins interact, a beta-galactosidase reporter gene (with an upstream *Gal4p* binding site) is activated. Nowadays, a library of haploid yeasts expressing bait-activation domain fusions can be mated sequentially with clones from a library of opposite mating type yeasts expressing prey-binding domain fusions (Uetz *et al.*, 2000; Ito *et al.*, 2001). In this set-up, the interaction causes expression of a gene (or genes) required for growth of the diploid on selective medium (e.g. *HIS3*, an auxotrophic marker). The development of other high-throughput strategies has allowed the use of this system to provide interaction networks for *Escherichia coli* bacteriophage Lambda (Bartel *et al.*, 1999) and *Helicobacter pylori* (Rain *et al.*, 2001). One important point to note though is that the datasets from the two global screens of yeast showed very few overlaps, indicating a high false negative rate for these approaches. This issue, and others, including standardisation of these experiments to allow comparison of interaction networks, are becoming important topics in proteomics (see Legrain P, p. 301).

Another technique for detecting interaction partners of chosen proteins is phage-display, in which short oligonucleotides are inserted within a gene encoding a coat protein of a bacteriophage, so that each viral peptide displays a different peptide sequence (for a review see Rodi and Makowski, 1999). Libraries of these phage are then screened against the protein of interest, to identify those peptides which bind the protein. Other display technologies are yeast display, in which the oligonucleotides are inserted into the genes for yeast surface proteins, and ribosome display (Hanes and Pluckthun, 1997), in which the translated protein emerges from the ribosome and can fold, but remains part of the ribosomal complex and associated with its mRNA, allowing its identification by

Copyright © 2001 John Wiley & Sons, Ltd.

sequencing. Phage display has been widely used for the selection and evolution of antibodies and this is the principal area to which ribosome display has so far been applied.

Surface plasmon resonance (SPR) requires the immobilisation of a protein of interest upon the sensor surface. Interacting proteins or peptides can then be identified from a complex mixture. The interaction is detected by the sensor and since the signal is linearly affected by the number of molecules bound (over a certain range) this allows quantitative or kinetic analyses to be performed. (For more detailed explanations of the technique, see **http://www.xantec.com/** and **http://www.biacore.com/biomol/principle.shtml**).

Peptide and protein domain arrays are the latest addition to the battery of tools for studying proteomes. In addition to protein expression profiling, they can be applied to the identification of interacting proteins. They have great potential for high-throughput use, but there is still a long way to go before they will become a widely used tool (see Taussig M, p. 298).

Using the sophisticated *in situ* techniques available today, proteins can be localised to highly specific regions of the cell and their movements can even be followed over time. The observation of co-localisation of proteins in such experiments can be used as an indicator of a potential interaction.

One approach for inferring protein-protein interactions uses structural predictions, based on homology to proteins of known structure with known binding partners. By modelling the structure of a novel member of a protein family against the known structures of other family members, it is possible to predict which protein partners the novel member will bind.

A further computational approach looks for protein pairs showing correlated sequence variations across species, indicating the presence of surfaces in these proteins that interact. Taking this concept further, are studies that look at the phylogenetic trees of two proteins, assuming that if they interact, it is likely that the trees will be similar.

There are also projects to identify 'functionally linked' proteins, which may or may not interact, but all the same are part of the same functional network in the cell (Marcotte *et al*., 1999). These can be proteins of known function that form part of the same pathway (commonly metabolic enzymes), proteins which show co-regulation of mRNA expression, proteins which are shown to be encoded by neighbouring genes across several species, or by a fused gene in one species, mainly in bacterial genomes (Enright *et al*., 1999), and proteins which share 'evolutionary profiles', which are their patterns of presence or absence across species.

Another important area in this field is the application of information extraction (IE) technology to biological research articles. A vast amount of data on protein-protein interactions resides in the published literature, which has never been entered into databases. Several groups have applied these approaches to gaining information on protein interactions (Marcotte *et al*., 2001, Blaschke and Valencia 2001).

Once we have assembled interaction networks, obvious uses are for the determination of gene functions and the better understanding of processes in the cell. This calls for the ability to compare networks and to integrate them with other sources of data. Two groups have already tried to apply this approach to yeast 'interactome' data. Schwikowski *et al*. (2000) conducted a global analysis of 2709 published interactions between yeast proteins. This resulted in a network of 2358 interactions among 1548 proteins. They saw that proteins of known function and cellular location tended to cluster together. In fact, 63% of the interactions occurred between proteins with a common functional assignment and 76% occurred between proteins found in the same subcellular compartment. Assigning possible functions to proteins based on the known functions of their interacting partners correctly predicted a functional category for 72% of the 1393 characterised proteins with at least one partner of known function.

Jeong *et al*. (2001) combined deletion phenotype data with the interaction map, to show that, even at this early stage (as demonstrated by the lack of overlap between the two global screens), essential genes tend to have a higher connectivity (that is they are involved in more interactions). They also found that random mutations, as modelled by randomly removing a protein from the network, do not affect the overall topology of the network. They propose then that the robustness of yeast against mutations comes from the organisation of its protein interactions. They also noted that the yeast network has the same topology as that for *Helicobacter pylori*, suggesting that there has been evolutionary selection of

Copyright © 2001 John Wiley & Sons, Ltd.

a common large-scale structure of interaction networks.

### Databases covering only experimentally verified interactions

The Fields' lab home page at the University of Washington provides access to the Yeast Protein Linkage Map (**http://depts.washington.edu/sfields/yplm/data/index.html**). This combines data from genome-wide two-hybrid screens, produced in collaboration with Curagen Corporation (Uetz *et al.*, 2000), with the results of a global analysis of 2,709 published interactions between yeast proteins (Schwikowski *et al.*, 2000). The data from the 2-hybrid screens is also available as the Curagen PathCalling Yeast Interaction Database (**http://portal.curagen.com/extpc/com.curagen.portal.servlet.Yeast**).

The Yeast Interacting Proteins Database (Ito *et al.*, 2001, **http://genome.c.kanazawa-u.ac.jp/Y2H/**) is available from the Ito lab webpages. The 'Workbench system for support of gene regulatory network construction' was developed for this data, by a Japanese company, INTEC Web and Genome Informatics Corporation (**http://genome.c.kanazawa-u.ac.jp/~webgen/webgen.html**). It operates as an application of Java 2 and is available from the site free of charge.

Hybrigenics has produced Protein Interaction Map (PIM) Rider™, a software platform based on reliable protein-protein interaction data, supported by links to databases with information on the genes, and to relevant literature (**http://pim.hybrigenics.com/**). There are two PIMRider™ resources that are freely available to academics, one for *Helicobacter pylori* protein interactions and one for the interactions between Human Immunodeficiency Virus proteins and human host proteins. These databases can be searched by gene, or protein name, or function comments. The table of results has links to the ProteinViewer entry or the Protein Interaction Map (PIM) of each protein. The Protein Viewer entry has information on the chosen protein and a table of all the proteins in its interaction network, followed by links to relevant literature, other databases and finally the sequence of the protein. The PIM is displayed using a Java tool, which is very similar to those used by ProNet, DIP and BIND, but incorporates more complexity (Figure 1).

ProNet combines a curational effort to gather information on published protein interactions, identified by two-hybrid screening, with data produced using Myriad Genetics' high-throughput yeast two-hybrid system. Currently the database only holds information on human proteins. Each protein has a 'home page', with its nucleotide and amino acid sequences, links to sequence, mapping and functional information, a graphic displaying motifs and domains, and a list of interacting proteins. From each protein home page, there is also a link to an interactive (Java) display of the interaction network that the protein is part of. The Pronet resource is freely available from DoubleTwist (**http://pronet.doubletwist.com**).

The Molecular INTeractions relational database (MINT, **http://160.80.34.232/mint/index.html**) is a collection of data manually retrieved from peer-reviewed journals about published protein-protein interactions, protein-nucleic acid interactions and multimeric complexes. The database has details on over 2000 interactions, from 45 organisms.

### Databases covering predicted (inferred) and experimentally verified interactions

The Database of Interacting Proteins (DIP, Xenarios *et al.*, 2000; **http://dip.doe-mbi.ucla.edu**) holds data on functional interactions between yeast proteins that have been inferred from a selection of computational approaches (Marcotte *et al.*, 1999), in addition to physical interactions proven by experiment. The criteria used to define functionally interacting proteins are; 'Related Metabolic Function' – proteins whose homologues have been shown experimentally to operate sequentially in a metabolic pathway; 'Related Phylogenetic Profiles' – proteins shown to have similar patterns of presence and absence across 20 fully sequenced genomes; the 'Rosetta Stone Method' – proteins whose homologues are fused into a single gene in another organism; and 'Correlated mRNA Expression' – genes showing correlated levels of mRNA expression across a series of assays. The study was initially run on yeast proteins, but now DIP holds data on just over 10,000 interactions, involving almost 6000 proteins, from 86 organisms.

Users can submit data on a new interaction to DIP, and edit or search existing data. Each interaction record has information on the proteins involved and the experimental evidence for the

Copyright © 2001 John Wiley & Sons, Ltd. 

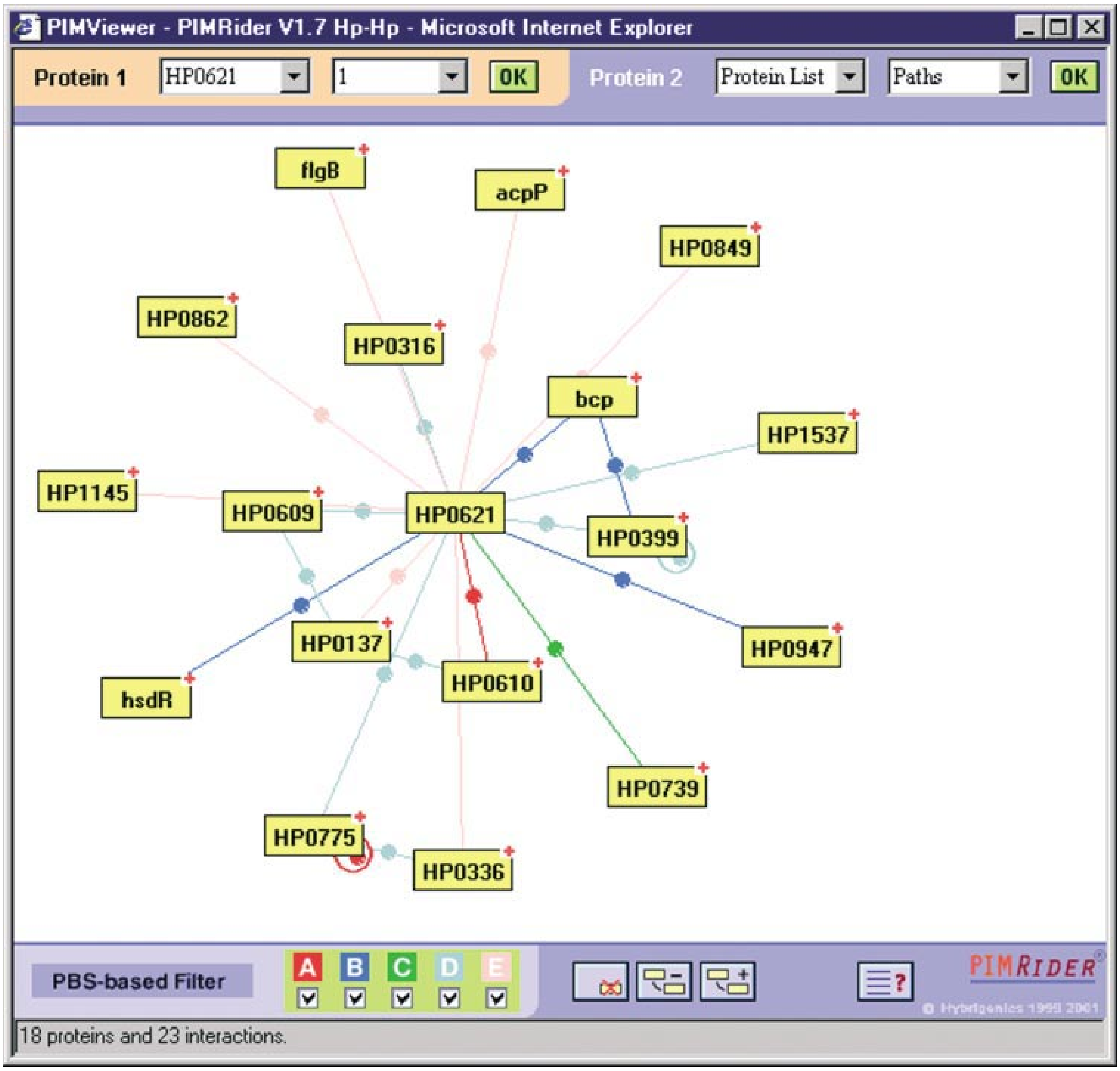

**Figure 1.** The PIMRider™ Java viewer showing the interaction network of HP0621 (mutS). Each yellow box is a gene, those with a red + sign on the top right corner have more neighbours, these can be retrieved by choosing that protein in the 'Protein 1' box and setting the number of neighbours you want to retrieve in the next box. Clicking OK will display that PIM. Using the Protein 1 and Protein 2 boxes it is possible to display the interaction between two chosen proteins. The PBS (PIM Biological Score) colour coded filters relate to the reliability of each interaction, with A being the best and D being the worst. E is used to denote those proteins that are very highly connected and are most likely false positives (or 'sticky prey'). This image is reproduced by kind permission of Hybrigenics S.A. © 2001 Hybrigenics S.A.

interaction, with links to published literature. The interaction network to which a protein of interest belongs can be viewed as a static image (Figure 2) or an interactive (java) depiction.

The Biomolecular Interaction Network Database (BIND, Bader *et al*., 2001) is designed to store full descriptions of interactions, molecular complexes and pathways (**http://www.bind.ca**). It currently contains information on just less than 6000 interactions, around 50 complexes, and 6 pathways, from 12 organisms, described using a defined data specification (Bader and Hogue, 2000). Users can

Copyright © 2001 John Wiley & Sons, Ltd.

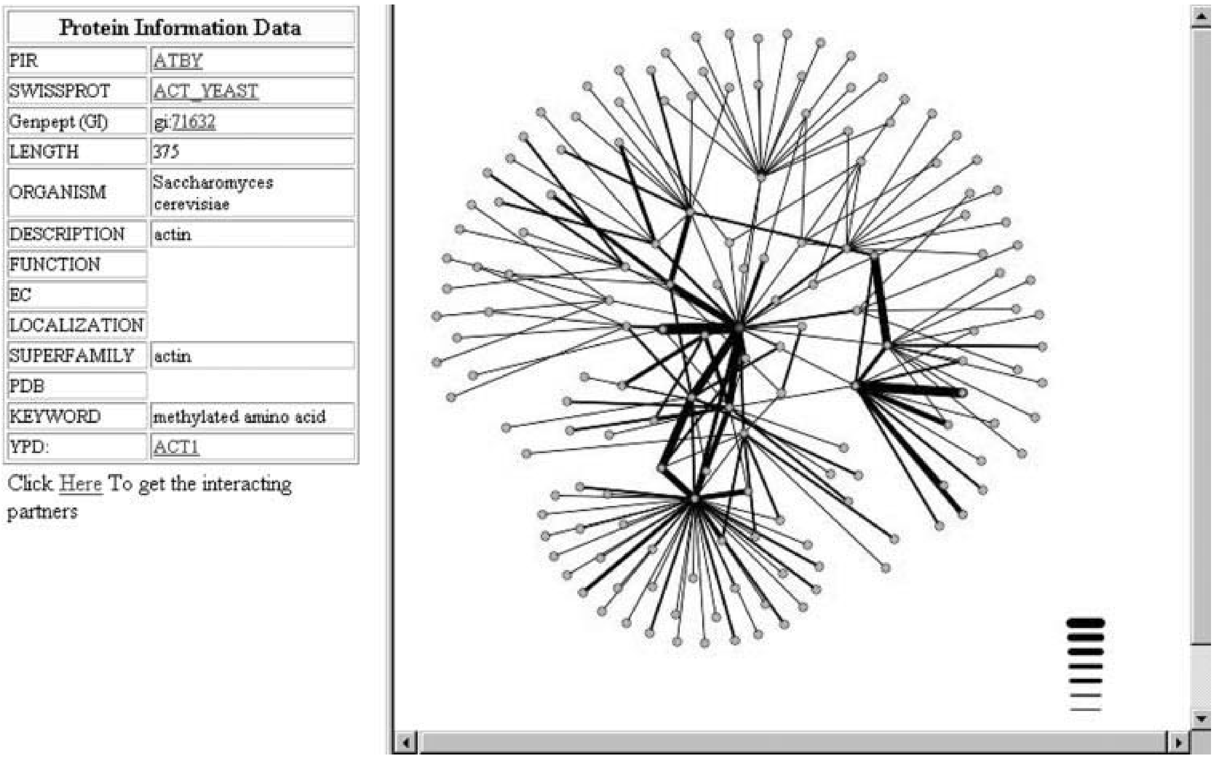

**Figure 2.** The static image format of the interaction network of yeast actin (ACT1) from DIP. Actin is shown at the centre of the network, and the window on the left shows details on actin. Clicking on any of the other proteins (nodes) in the network causes the left window to show details of that gene. The thickness of the lines joining the proteins indicates how many methods confirmed the linkage between those two proteins, the thicker the line, the more confidence there is in that linkage. This is image reproduced by kind permission of Ioannis Xenarios 2

add or change entries, or search or browse the existing data. A typical interaction record has information on the proteins or molecules making the interaction (name, ID codes and links to databases, origin and organism), an option to visualise the interaction (using a Java applet), and other information, such as publications relating to the interaction, the experimental approach used, and 3D structure information. The BIND site also has a useful listing of databases related to protein interactions.

### Organism specific databases

The *Drosophila* Protein Interaction Map (PIM) database (**http://cmmg.biosci.wayne.edu/finlab/PIMdbv01.htm**) is currently available as an HTML table, which can only be searched using the text-searching tool of the Internet browser. However, an Oracle 8i database is under development.

The *Caenorhabditis elegans* interaction mapping project is provided by the Vidal lab at the Dana Farber Cancer Institute (**http://vidal.dfci.harvard.edu/**). This project was started using 29 proteins involved in vulval development.

### Tools: structure-based prediction

The Protein-Protein Interaction Server (**http://www.biochem.ucl.ac.uk/bsm/PP/server/**) is a tool for analysing the protein-protein interface between any two polypeptide chains in the three dimensional structure of a protein complex. Users can submit the coordinates of a chosen protein structure and then view tables describing the nature of the protein-protein interface between the two chosen chains.

iSPOT is a resource provided by the same group that runs MINT, this tool is designed to look at the structure of the interfaces between proteins and was

Copyright © 2001 John Wiley & Sons, Ltd.

initially designed for studying the SH3 domain family (**http://cbm.bio.uniroma2.it/iSPOT**). The tool can be used to predict the binding partners of a related protein, by modelling its structure against those known for other family members. The tool has recently been modified for application to the PDZ domain family and MHC class I molecules.

### Tools: information extraction

The Valencia group have designed the Suiseki Information Extraction System, to collect information on experimentally verified protein-protein interactions from text records such as Medline abstracts. A description of the system and the results of several analyses are available at: **http://www.pdg.cnb.uam.es/suiseki/index.html** (see also Blaschke and Valencia, 2001).

### References

Bader GD, Donaldson I, Wolting C, Ouellette BF, Pawson T, Hogue CW. 2001. BIND – The Biomolecular Interaction Network Database. *Nucleic Acids Res* **29**(1): 242–245.

Bader GD, Hogue CW. 2000. BIND – a data specification for storing and describing biomolecular interactions, molecular complexes and pathways. *Bioinformatics* **16**(5): 465–477.

Bartel PL, Roecklein JA, SenGupta D, Fields S. 1996. A protein linkage map of *Escherichia coli* bacteriophage T7. *Nat Genet* **12**(1): 72–77.

Blaschke C, Valencia A. 2001. Can bibliographic pointers for known biological data be found automatically? Protein interactions as a case study. *Comp Funct Genom* **2**(4): 196–206.

Enright AJ, Iliopoulos I, Kyrpides NC, Ouzounis CA. 1999. Protein interaction maps for complete genomes based on gene fusion events. *Nature* **402**: 86–90.

Fields S, Song OK. 1989. A novel genetic system to detect protein-protein interactions. *Nature* **340**(6230): 245–246.

Hanes J, Pluckthun A. 1997. In vitro selection and evolution of functional proteins by using ribosome display. *Proc Natl Acad Sci U S A* **94**(10): 4937–4942.

Ito T, Chiba T, Ozawa R, Yoshida M, Hattori M, Sakaki Y. 2001. A comprehensive two-hybrid analysis to explore the yeast protein interactome. *Proc Natl Acad Sci U S A* **98**(8): 4277–4278.

Jeong H, Mason SP, Barabasi AL, Oltvai ZN. 2001. Lethality and centrality in protein networks. *Nature* **411**(6833): 41–42.

Marcotte EM, Pellegrini M, Thompson MJ, Yeates TO, Eisenberg D. 1999. A combined algorithm for genome-wide prediction of protein function. *Nature* **402**: 83–86.

Marcotte EM, Xenarios I, Eisenberg D. 2001. Mining literature for protein-protein interactions. *Bioinformatics* **17**(4): 359–363.

Rain JC, Selig L, De Reuse H, *et al.* 2001. The protein-protein interaction map of *Helicobacter pylori*. *Nature* **409**(6817): 211–215.

Rodi DJ, Makowski L. 1999. Phage-display technology – finding a needle in a vast molecular haystack. *Curr Opin Biotechnol* **10**: 87–93.

Schwikowski B, Uetz P, Fields S. 2000. A network of protein-protein interactions in yeast. *Nat Biotechnol* **18**(12): 1257–1261.

Uetz P, Giot L, Cagney G, *et al.* 2000. A comprehensive analysis of protein-protein interactions in *Saccharomyces cerevisiae*. *Nature* **403**(6770): 623–627.

Xenarios I, Rice DW, Salwinski L, Baron MK, Marcotte EM, Eisenberg D. 2000. DIP: the Database of Interacting Proteins. *Nucleic Acids Res* **28**: 289–291.

---

Some of the sites reviewed will already be known to you but perhaps their content will be less well-known. The Website Review is intended to help you discover new sites of interest, but also to provide a rapid and convenient means of revealing what you always knew was there but never had the time or inclination to look at. These articles are a personal critical analysis of the Websites. If you have any information about sites you think are worthy of being more widely known, the Managing Editor would be pleased to hear from you.

Copyright © 2001 John Wiley & Sons, Ltd.
